# Develop and validate nomogram to predict cancer-specific survival for patients with testicular yolk sac tumors

**DOI:** 10.3389/fpubh.2022.1038502

**Published:** 2022-10-17

**Authors:** Maoxian Li, Jinkui Wang, Jinfeng Li, Yongbo Zhang, Xing Zhao, Yang Lin, Changkai Deng, Fulin Li, Qiang Peng

**Affiliations:** ^1^Department of Pediatric Surgery, Chengdu Women's and Children's Central Hospital, School of Medicine, University of Electronic Science and Technology of China, Chengdu, China; ^2^Department of Urology, Ministry of Education Key Laboratory of Child Development and Disorders, National Clinical Research Center for Child Health and Disorders, China International Science and Technology Cooperation Base of Child Development and Critical Disorders, Children's Hospital of Chongqing Medical University, Chongqing, China

**Keywords:** testicular, yolk sac tumors, cancer-specific survival, SEER, nomogram

## Abstract

**Purpose:**

Testicular yolk sac tumor (TYST) is a rare malignant germ cell tumor that mainly occurs in young men. Due to the low incidence of yolk sac tumors, there is a lack of prospective cohort studies with large samples. We aimed to develop a nomogram to predict cancer-specific survival (CSS) in patients with TYST.

**Materials and methods:**

Patient information was downloaded from the Surveillance, Epidemiology and End Results (SEER) database. We enrolled all patients with TYST from 2000 to 2018, and all patients were randomly divided into a training set and a validation set. Univariate and multivariate Cox proportional hazards regression models were used to identify independent risk factors for patients. We constructed a nomogram based on the multivariate Cox regression model to predict 1-, 3-, and 5-year CSS in patients with TYST. We used a series of validation methods to test the accuracy and reliability of the model, including the concordance index (C-index), calibration curve and the area under the receiver operating characteristic curve (AUC).

**Results:**

619 patients with TYST were enrolled in the study. Univariate and multivariate Cox regression analysis showed that age, T stage, M stage and chemotherapy were independent risk factors for CSS. A nomogram was constructed to predict the patient's CSS. The C-index of the training set and the validation set were 0.901 (95%CI: 0.859–0.847) and 0.855 (95%CI: 0.865–0.845), respectively, indicating that the model had excellent discrimination. The AUC showed the same results. The calibration curve also indicated that the model had good accuracy.

**Conclusions:**

In this study, we constructed the nomogram for the first time to predict the CSS of patients with TYST, which has good accuracy and reliability and can help doctors and patients make clinical decisions.

## Introduction

Testicular yolk sac tumor (TYST) is a rare malignant germ cell tumor with an incidence of <1% (1). The disease is often accompanied by elevated serum alpha-fetoprotein (AFP) (1–4). TYST accounts for 11–18% of germ cell tumors and 10–44% of non-seminoma germ cell tumors (4). Due to its high degree of malignancy, insidious onset and rapid progression, TYST seriously threatens the survival of patients (2, 3). Surgical resection of the primary tumor is a key factor in treatment. Targeted therapy with chemotherapy, retroperitoneal lymph node dissection and distant metastasis have made widely disseminated testicular germ cell tumors treatable (5). Due to the low incidence of TYST, there is a lack of studies on TYST with large samples. The related factors affecting the prognosis of TYST are still unclear.

Previous studies have found that the maximum diameter of the tumor, low echo and low blood flow signal under ultrasound can predict the degree of malignancy of testicular tumors and thus predict the prognosis of patients (6). In addition, a study on germ cell tumors (including testicular and ovarian yolk sac tumors) found that patients with slow AFP decline and long half-life after surgery tended to have poor long-term prognoses (7). Unfortunately, the predictive value of these factors remained unclear. Physicians often make empirical judgments on the prognosis of TYST patients based on the patient's age, clinical stage, surgical condition and chemotherapy, but this method cannot intuitively and quantitatively predict patients' survival.

The National Cancer Institute's Surveillance, Epidemiology, and End Results (SEER) database provides high confidence data on many cancer patients since 1973. The nomogram prediction model has been widely used to predict the prognosis of a variety of solid tumors and is considered to be one of the most accurate ways to predict tumors (8), including UISS (9) and SSIGN (10). However, for the above model, there are no relevant reports of these clinical variables for TYST cases. This study retrospectively analyzed the data of TYST patients from the SEER database to explore the clinicopathological features affecting their prognosis. According to these features, a nomogram was constructed to predict the cancer-specific survival (CSS) of TYST patients.

## Patients and methods

### Data source and data extraction

Patient data were downloaded from the SEER database to identify all patients with TYST. The SEER database is the US national cancer database that includes patients from 18 cancer registries, covering about 30% of the US population. The clinicopathological information of the patients was publicly available. Since the patients' personal information could not be identified, ethical approval and informed consent were not required for our study.

**Figure 1:**
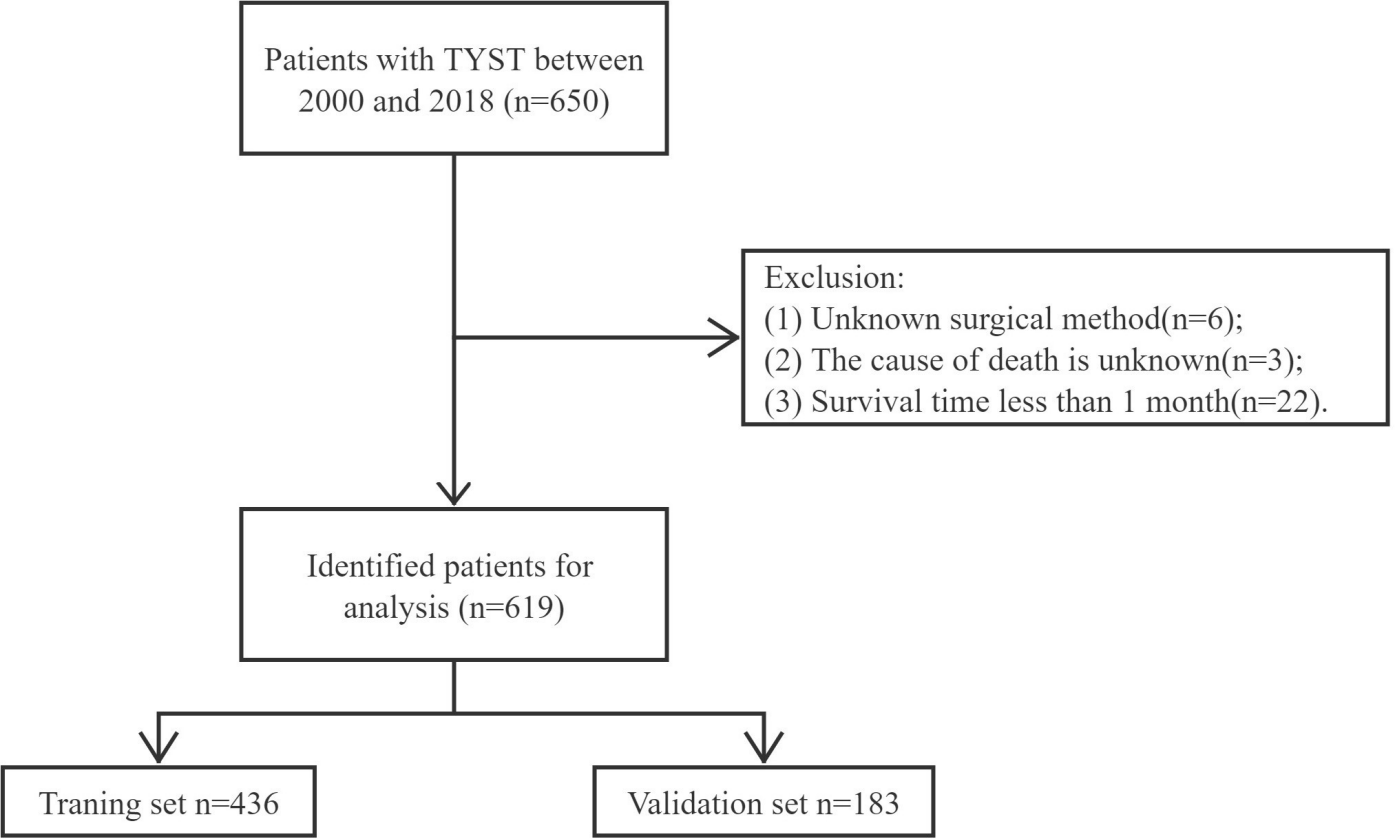
Flowchart for inclusion and exclusion of all patients with TYST.

We collected demographic information (age, marital status, race), tumor information (laterality, tumor stage), treatment information (surgery, radiotherapy, chemotherapy), and follow-up information (survival time, survival status, cause of death). Inclusion criteria: (1) pathological diagnosis of TYST; (2) Complete follow-up information. Exclusion criteria: (1) Unknown surgical method; (2) The cause of death is unknown; (3) Survival time <1 month. The inclusion and exclusion process of patients is shown in Figure 1.

Patients were classified as white, black, and other (American Indian/AK Native, Asian/Pacific Islander). The patient's marital status was classified as married, single, and divorced. The laterality of the tumor is classified as left, right, and bilateral. Surgery was classified as non-surgery and excision of testicle. According to whether the patient received radiotherapy, it was classified as yes or no. According to whether the patient received chemotherapy, it was classified as yes or no. Causes of death include death from cancer or death from other reasons.

### Nomogram development and validation

All patients were randomized into a training set (70%) and a validation set (30%). We first used univariate Cox regression models in the training set to analyze the factors affecting the patients' CSS. Subsequently, the factors identified by univariate Cox regression analysis were included in the multivariate Cox regression model to screen for independent risk factors affecting patients' CSS. We developed a nomogram based on multivariate Cox regression model to predict CSS in patients with TYST. Then, the concordance index (C-index), the calibration curve, and the area under the receiver operating characteristic curve (AUC) were used to validate the accuracy and discrimination of the model in the training set and the validation set.

### Clinical utility

We use decision curve analysis (DCA) to assess the clinical potential practical value of the model. DCA is a new algorithm based on calculating net returns under different thresholds. In addition, we calculated the optimal cut-off value based on the receiver operating characteristic curve (ROC) based on the risk scores of all patients. Then, according to the cut-off value, we developed a risk stratification system that divides patients into high-risk and low-risk groups. We used the log-rank test and the Kaplan-Meier (K-M) curve to test the survival differences of patients in different risk groups.

### Statistical analysis

SPSS 26.0 was used for all statistical analyses. R software 4.1.0 was used to develop and validate the nomogram. Categorical variable data were described by frequency (%), and differences between groups were compared by the chi-square test. Data of continuous variables were described by means and standard deviations, and differences between groups were analyzed by student's *t*-test or non-parametric U test. The Cox regression model analyzed the survival factors of patients. The log-rank test was used to compare differences in survival among different groups of patients. A *p* value of < 0.05 was considered statistically significant.

## Result

### Clinical features

In total, we enrolled 619 patients with TYST. Patients were randomly assigned to the training set (*n* = 436) and the validation set (*n* = 183). The mean age of the patients was 24.2 ± 17.0 months. There were 530 (85.6%) white and 131 (21.2%) married patients. There were 300 (48.5%) tumors located in the left testis, 327 (52.8%) tumors confined to the primary site, 275 (44.4%) tumors were stage I, 36 (5.82%) tumors were stage II, and 158 (25.5%) tumors were stage III. The 563 (91.0%) patients underwent orchiectomy, 15 (2.4%) patients received radiotherapy, and 270 (43.6%) patients received chemotherapy. The survival time of all patients was 83.6 ± 67.1 months. The information of patients in the training and validation sets is shown in Table 1, and there is no significant difference between the two groups.

**Table 1:** Clinical characteristics of patients with TYST.

	**ALL**	**Training cohort**	**Validation cohort**	
	***N* = 619**	***N* = 436**	***N* = 183**	***p***
Age	24.2 (17.0)	23.9 (17.6)	24.9 (15.8)	0.504
Race				0.875
White	530 (85.6%)	375 (86.0%)	155 (84.7%)	
Black	33 (5.33%)	22 (5.05%)	11 (6.01%)	
Other	56 (9.05%)	39 (8.94%)	17 (9.29%)	
Marital				0.495
Married	131 (21.2%)	93 (21.3%)	38 (20.8%)	
Singled	439 (70.9%)	305 (70.0%)	134 (73.2%)	
Divorced	49 (7.92%)	38 (8.72%)	11 (6.01%)	
Laterality				0.282
Left	300 (48.5%)	219 (50.2%)	81 (44.3%)	
Right	282 (45.6%)	194 (44.5%)	88 (48.1%)	
Unknown	37 (5.98%)	23 (5.28%)	14 (7.65%)	
AJCC				0.148
I	275 (44.4%)	202 (46.3%)	73 (39.9%)	
II	36 (5.82%)	23 (5.28%)	13 (7.10%)	
III	158 (25.5%)	115 (26.4%)	43 (23.5%)	
Unknown	150 (24.2%)	96 (22.0%)	54 (29.5%)	
T				0.522
T1	277 (44.7%)	200 (45.9%)	77 (42.1%)	
T2	102 (16.5%)	76 (17.4%)	26 (14.2%)	
T3	37 (5.98%)	26 (5.96%)	11 (6.01%)	
T4	16 (2.58%)	11 (2.52%)	5 (2.73%)	
TX	187 (30.2%)	123 (28.2%)	64 (35.0%)	
M				0.122
M0	359 (58.0%)	259 (59.4%)	100 (54.6%)	
M1	139 (22.5%)	101 (23.2%)	38 (20.8%)	
MX	121 (19.5%)	89 (20.5%)	32 (17.3%)	
Surgery				1.000
No	56 (9.05%)	39 (8.94%)	17 (9.29%)	
Yes	563 (91.0%)	397 (91.1%)	166 (90.7%)	
Radiotherapy				0.777
No	604 (97.6%)	426 (97.7%)	178 (97.3%)	
Yes	15 (2.42%)	10 (2.29%)	5 (2.73%)	
Chemotherapy				0.766
No	349 (56.4%)	248 (56.9%)	101 (55.2%)	
Yes	270 (43.6%)	188 (43.1%)	82 (44.8%)	
Regional nodes examined				0.128
No	551 (89.0%)	394 (90.4%)	157 (85.8%)	
Yes	68 (11.0%)	42 (9.63%)	26 (14.2%)	
Regional nodes positive				0.273
No	595 (96.1%)	422 (96.8%)	173 (94.5%)	
Yes	24 (3.88%)	14 (3.21%)	10 (5.46%)	
CSS				0.216
Dead	68 (11.0%)	43 (9.86%)	25 (13.7%)	
Alive	551 (89.0%)	393 (90.1%)	158 (86.3%)	
Survival months	83.6 (67.1)	84.7 (66.1)	80.9 (69.4)	0.531
Status				0.621
Dead	93 (15.0%)	63 (14.4%)	30 (16.4%)	
Alive	526 (85.0%)	373 (85.6%)	153 (83.6%)	

AJCC, American Joint Committee on Cancer; T, Tumor; M, Metastasis.

### Univariate and multivariate cox regression analysis

Univariate and multivariate Cox regression analyses were used to explore the prognostic factors for CSS. Specifically, we first used univariate Cox regression analysis to identify factors associated with CSS. The results showed that age, laterality, T stage, M stage, surgery, radiotherapy, and chemotherapy were the factors affecting CSS. These seven factors were then included in a multivariate Cox regression analysis to identify independent risk factors. Finally, we found that age, T stage, M stage, laterality, and chemotherapy were independent predictors of CSS in patients. The results of univariate and multivariate Cox regression analysis are shown in Table 2.

**Table 2:** Univariate and multivariate analyses of CSS in training set.

	**Univariate**			**Multivariate**		
	**HR**	**95%CI**	***P***	**HR**	**95%CI**	***P***
Age	1.04	1.03–1.06	<0.001	1.027	1.01–1.05	0.003
Race						
White	1.22	0.3–5.06	0.782			
Black	0.98	0.35–2.75	0.974			
Other	0	0-Inf	0.997			
Marital						
Married	0.94	0.45–1.98	0.877			
Singled	2.77	0.85–8.99	0.09			
Divorced	0.64	0.08–5.07	0.675			
Laterality						
Left						
Right	8.74	4.26–17.97	<0.001	0.658	0.317–1.365	0.261
Unknown	0.76	0.38–1.5	0.424	3.1	1.405–6.839	0.005
T						
T1						
T2	1.53	0.46–5.07	0.49	2.147	0.622–7.413	0.227
T3	6.73	2.33–19.39	<0.001	3.377	1.113–10.248	0.032
T4	17.06	5.55–52.44	<0.001	5.498	1.702–17.759	0.004
TX	4.34	1.94–9.7	<0.001	3.851	1.554–9.543	0.004
M						
M0						
M1	14.54	6.05–34.95	<0.001	4.209	1.586–11.172	0.004
MX	3.87	1.4–10.68	0.009	1.382	0.42–4.555	0.595
AJCC						
I						
II	2.13	0.24–19.07	0.499			
III	16.24	5.73–46.04	<0.001			
Unknown	4.01	1.26–12.79	0.019			
Surgery						
No						
Yes	0.14	0.07–0.26	<0.001			
Radiotherapy						
No						
Yes	4.06	1.46–11.34	0.007			
Chemotherapy						
No						
Yes	7.32	3.41–15.72	<0.001	2.491	1.041–5.958	0.04

### Construction of 1-, 3-, and 5-year CSS nomogram

**Figure 2:**
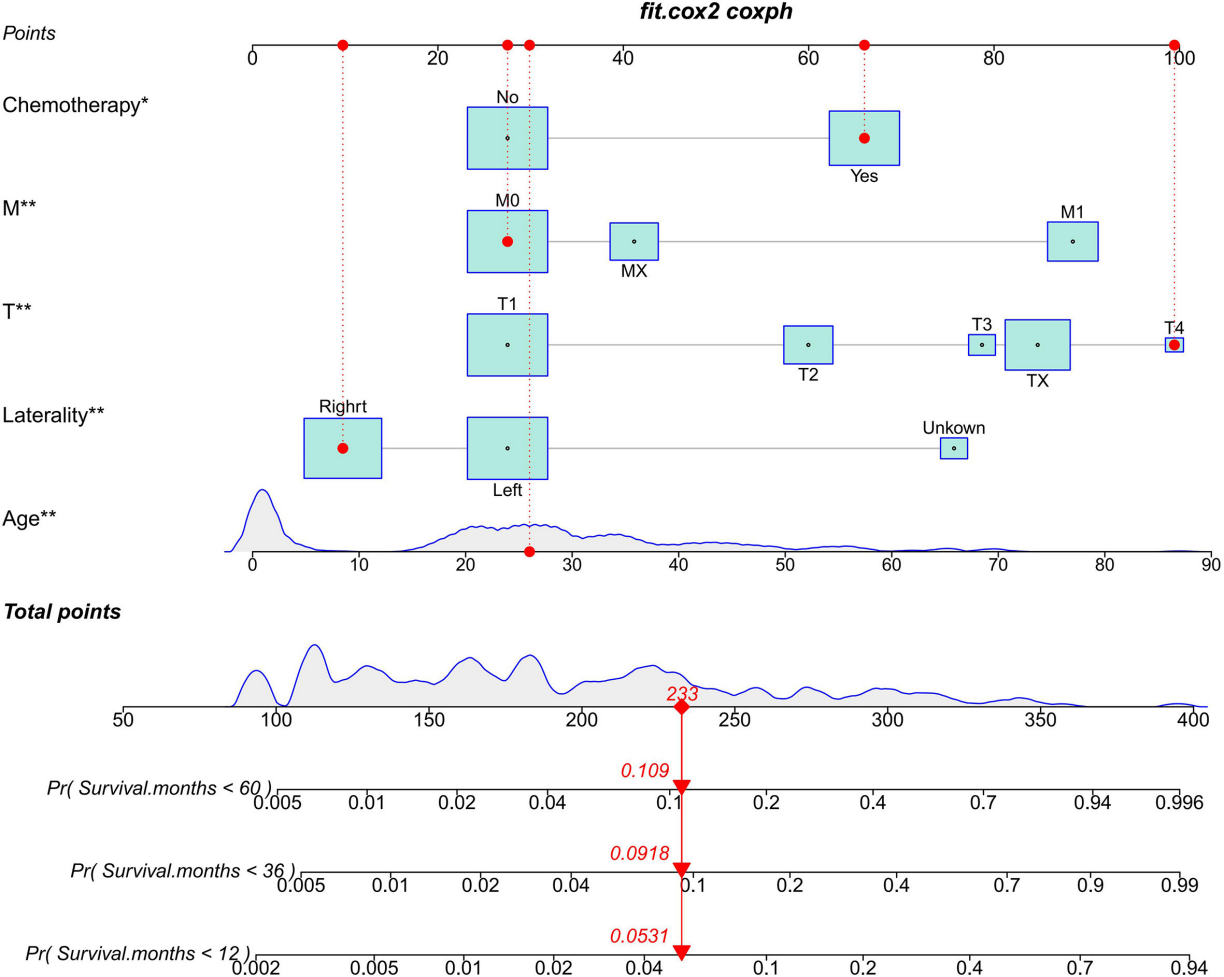
The nomogram predicts CSS in patients with TYST at 1-, 3-, and 5-year. * *P* < 0.05, ** *P* < 0.01.

Based on the multivariate Cox regression model, we constructed a nomogram for predicting CSS in TYST patients. Specifically, the model includes age, T stage, M stage, laterality, and chemotherapy. As shown in Figure 2, patients' risk of death increased with age. The higher the T stage and M stage, the higher the risk of death. Patients with left-sided tumors have a lower survival rate than those with right-sided tumors.

### Validation of nomogram

**Figure 3:**
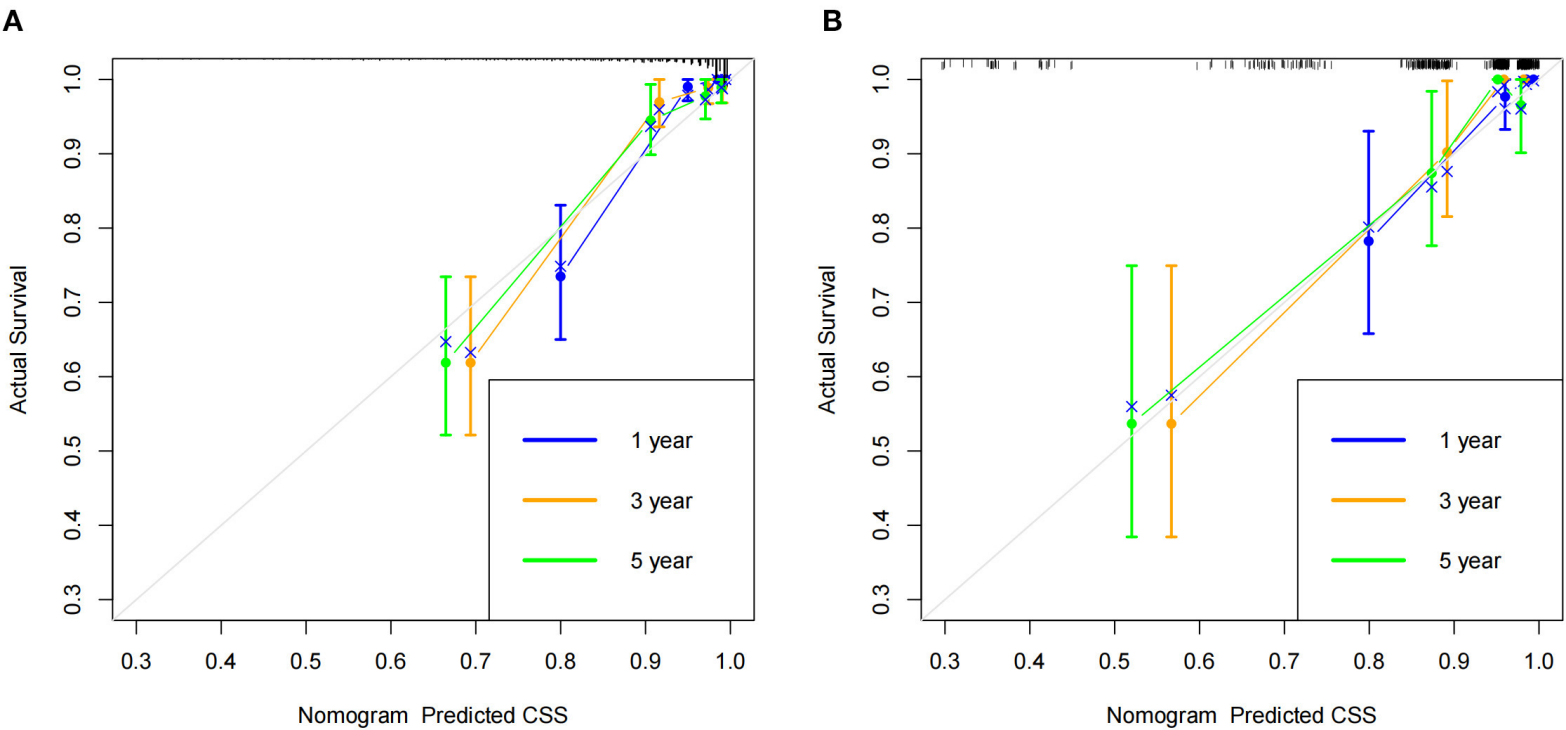
Calibration curve of the nomogram. **(A)** Calibration curves of 1-, 3-, and 5-year CSS in the training set; **(B)** Calibration curves of 1-, 3-, and 5-year CSS in the validation set.

**Figure 4:**
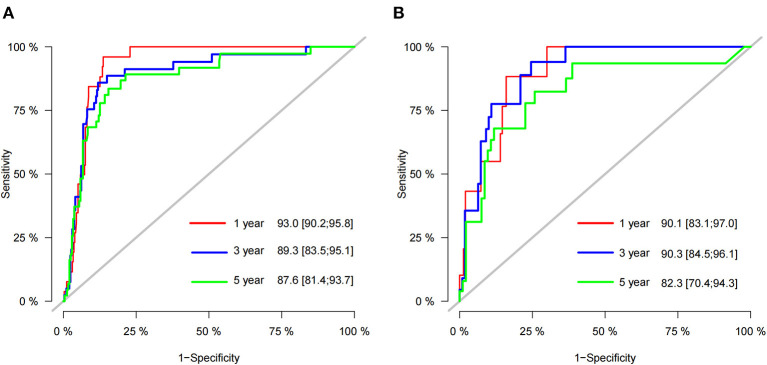
AUC for predicting 1-, 3-, and 5-year CSS in the training set **(A)** and validation set **(B)**.

According to the established nomogram, we first used the C-index to validate the model's accuracy. The range of C-index is between 0.5 and 1, and a high C-index indicates a high discrimination of the model. The C-index of the training and validation sets were 0.886 (95%CI: 0.831–0.941) and 0.876 (95%CI: 0.829–0.923), respectively, indicating the high discrimination of the model. In addition, the calibration curve was used to validate the model's accuracy. As shown in Figure 3, in the training and validation sets, the predicted and observed values were highly consistent, indicating the high accuracy of the nomogram. In addition, AUC was used to validate the discrimination of the model (Figure 4). Compared with the C-index, the time-dependent AUC can more clearly show the trend of model discrimination over time. In the training set, the AUC of nomogram were 93.0, 89.3 and 87.6 at 1-, 3- and 5-year, respectively. In the validation set, the AUC of nomogram were 90.1, 90.3, and 82.3 at 1-, 3- and 5-year, respectively. It shows that the model has high discrimination.

### Clinical application of the nomogram

**Figure 5:**
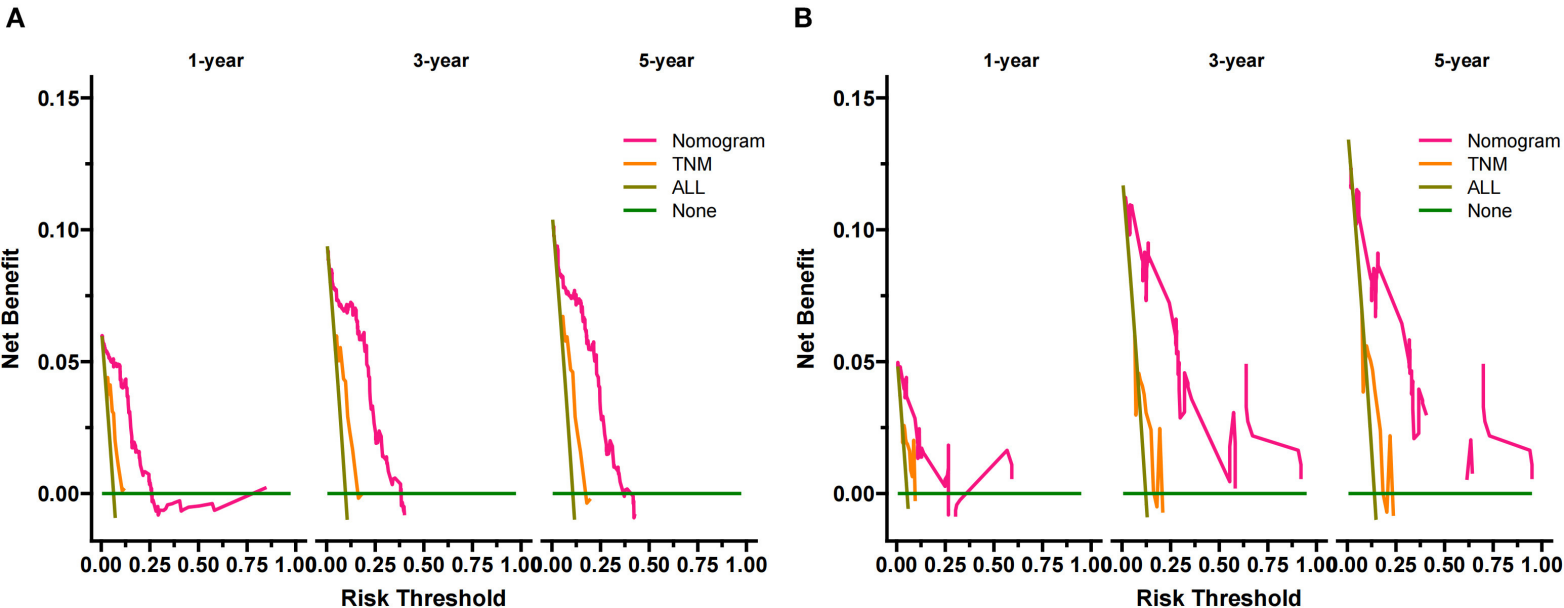
DCA of the nomogram in the training set **(A)** and the validation set **(B)**. The Y-axis represents a net benefit, and the X-axis represents threshold probability. The green line means no patients died, and the dark green line means all patients died. When the threshold probability is between 0 and 60%, the net benefit of the model exceeds all deaths or none.

**Figure 6:**
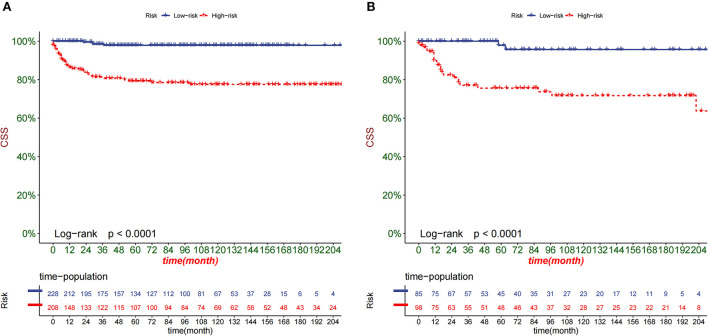
Kaplan-Meier curves of children in the low-risk and high-risk groups in the training set **(A)** and validation set **(B)**.

DCA is used to validate the practical value of the model. As shown in Figure 5, the DCA of the nomogram was superior to the traditional TNM staging in both the training and validation sets. In addition, according to the nomogram, we construct a new risk stratification system. We calculated the risk value for each patient and then divided the patients into a high-risk group (total score ≥94.5) and a low-risk group (total score <94.5) using the optimal cut-off value of the ROC. In the high-risk group, patients' 1-, 3- and 5-year survival rates were 87.5, 79.6, and 78.1%, respectively. The patients' 1-, 3-, and 5-year survival rates were 100.0, 98.8, and 97.9% in the low-risk group. In both the training and validation sets, patients in the high-risk group had significantly lower survival than those in the low-risk group (Figure 6).

## Discussion

TYST has two peak onset periods, within 2 years after birth and > 15 years of age. With the development of medical technology, the overall survival rate of TYST has gradually increased to 90% (1, 11). To further improve the quality of life of TYST patients and accurately predict the prognosis value of TYST patients concerned. In clinical practice, in addition to TNM staging, there is a lack of a model that can accurately predict the prognosis of patients with TYST.

Nomogram is a data-based graphical computing tool that predicts the risk of developing a disease by incorporating the American Joint Committee on Cancer (AJCC) TMN staging system and other important risk factors associated with prognosis (12, 13). Compared with traditional TMN staging, nomogram has better accuracy in predicting prognosis and can better provide advice and help for clinicians in diagnosis and treatment (14). To our knowledge, no prognostic nomogram of patients with TYST has been reported. Due to the low incidence of testicular yolk sac tumor, it isn't easy to collect a large sample size for a single-center study to obtain reliable conclusions. Therefore, it is very important to establish a reliable and accurate prognosis model for TYST. SEER database covers 18 countries and regions. The biggest advantage of the SEER database is that it can obtain clinical data with large samples, which is especially helpful for studying rare cases (4). This study collected data from the SEER database, effectively avoiding the insufficient sample size of TYST and single clinical data.

In this study, we developed and validated a novel nomogram to accurately predict the specific survival of patients with TYST. It is well known that the chance of genetic mutations causing cancer increases with age. Studies have shown that age plays a key role in the survival of various cancers (15, 16). A prediction study on non-spermatogonial cell tumors found that the survival rate of adolescent patients was higher than that of adults (17). Previous studies have reported that post-pubertal TYST often has a poor prognosis (18). Talerman et al. also found that the prognosis of adult testicular yolk sac tumor was worse than that of infants (19). Our study identified age as an independent risk factor for testicular yolk sac tumor-specific survival. In other words, the CSS rate of TYST patients decreased gradually with age.

The TNM staging system is often used to evaluate various malignant tumors, helps judge the prognosis of malignant tumors, and guides clinicians to adopt better treatment strategies (20, 21). Eighty to 85% of patients with TYST were stage I, confined to the primary part of the tumor. Radical testicular resection through the inguinal region often has a good prognosis (1, 3, 22). In this study, the operation was also found to be a specific protective factor for long-term survival. This study found that the T and M stages are independent risk factors for predicting the specific survival of patients with TYST. The prognosis is often poor for the high-level T and M stages, consistent with previous studies' results (23). In our prediction model, the *n* stage was not an independent risk factor for patients with testicular yolk sac prognosis. For patients with retroperitoneal metastasis, retroperitoneal lymph node dissection is used to improve the prognosis of patients. However, this study found that 68 patients underwent retroperitoneal lymph node dissection (RPLND), and only 24 were positive. Standard retroperitoneal lymphadenectomy involves the removal of all lymphoid tissue from the hilum to 2cm distal to the common iliac artery bifurcation and from both sides to the level of the ureter. All large vessels need to be skeletal. RPLND is prone to erectile dysfunction, lymphatic leakage (lymphedema, chylous ascites), surgical injury (most commonly vascular injury and ureteral injury), surgical infection (lung, incision), intestinal obstruction and other complications (24, 25). Besides, infertility was the major long-term toxicity associated with RPLND (24). Therefore, routine retroperitoneal lymphadenectomy is not recommended for children with TYST (1, 5, 26), and even RPLND is mainly resection of retroperitoneal mass rather than radical RPLND. This study showed that retroperitoneal lymph node dissection did not significantly improve long-term survival. Accordingly, we do not recommend RPLND for TYST patients without a high risk of recurrence. This can improve a patient's quality of life without reducing the long-term specific survival rate.

Although postoperative radiotherapy has a good effect in treating various tumors and has been included in various cancer guidelines, TYST is not sensitive to radiotherapy, and it was found in this study that postoperative radiotherapy could not improve the cancer-specific survival rate of TYST patients. In addition, this study found that chemotherapy is the main risk factor affecting the prognosis of TYST, and chemotherapy patients often indicate a poor prognosis. Geng et al. reported similar results when they established a nomogram to predict the survival rate of yolk sac tumors (4). According to the 2015 European guidelines for Urology treatment, chemotherapy is not recommended for patients in stage I without high-risk factors. Chemotherapy is recommended for patients unwilling to undergo surveillance or those with high-risk stage I (27). Our study showed doctors' importance in identifying patients with high-risk stage I TYST to avoid unnecessary chemotherapy. So 80–95% of TYST patients could be protected from the toxic effects of chemotherapy (24). Besides, chemotherapy treatment for high-stage patients might not predict a good prognosis. At the same time, it was impossible to further investigate the confounders due to the lack of details on chemotherapy and surgery.

Finally, the newly constructed Nomogram model for predicting the CSS rate of TYST patients includes multiple factors such as age at diagnosis, TM stage, and chemotherapy, which is convenient for clinical information collection. Although the International Germ Cell Cancer Collaborative Group (IGCCCG) is commonly used to predict the prognosis of metastatic non-spermatogenial germ cell tumor, it targets all testicular cancers and predict the individual 3-year progression-free survival rate of patients (28). Our present developed nomogram depending on SEER can accurately predict CSS at 1, 3, and 5 years in patients with TYST.

However, this study still has some limitations. First, although the SEER database collected clinical data of patients from multiple medical centers in the United States, much detailed clinical information (the patient's family history, laboratory test results (AFP and LDH), maximum tumor diameter, surgical scope and intraoperative conditions, pathological history, chemotherapy regimen, recurrence and metastasis) were not recorded. This clinical information has been proven to be related to the prognosis of TYST (1, 6, 11, 28, 29), and adding such information can further improve the model's accuracy. Second, this study used an internal validation method to validate the nomogram model, and an external cohort was needed to further validate the accuracy of the model. Finally, since this study is retrospective, there must be selection bias. Therefore, prospective studies are needed to further validate the model.

## Conclusion

In the study, we explored prognostic factors in TYST patients. The results showed that the patient's age, T stage, M stage and chemotherapy were independent risk factors affecting the CSS of the patients. We constructed a nomogram to predict the CSS of TYST patients. After internal validation, the nomogram has been proven to have good accuracy and reliability. This predictive tool can help physicians predict the prognosis of TYST patients and develop appropriate monitoring and follow-up plans.

## Data availability statement

Publicly available datasets were analyzed in this study. This data can be found here: The SEER data analyzed in this study is available at https://seer.Cancer.gov/.

## Author contributions

ML and JW designed the study. JW, JL, YZ, and XZ collected and analyzed the data. ML drafted the initial manuscript and reviewed and edited the article. YL, CD, FL, and QP revised the article critically. All authors approved the final manuscript, contributed to the article, and approved the submitted version.

## Conflict of interest

The authors declare that the research was conducted in the absence of any commercial or financial relationships that could be construed as a potential conflict of interest.

## Publisher's note

All claims expressed in this article are solely those of the authors and do not necessarily represent those of their affiliated organizations, or those of the publisher, the editors and the reviewers. Any product that may be evaluated in this article, or claim that may be made by its manufacturer, is not guaranteed or endorsed by the publisher.

## References

[1] Gilligan T Lin DW Aggarwal R Chism D Cost N Derweesh IH. Testicular Cancer, Version 2.2020, NCCN clinical practice guidelines in oncology. J Natl Compr Canc Netw. (2019) 17:1529–54. 10.6004/jnccn.2019.0100 31805523

[2] Jarvis H Cost NG Saltzman AF. Testicular tumors in the pediatric patient. Semin Pediatr Surg. (2021) 30:151079. 10.1016/j.sempedsurg.2021.151079 34412887

[3] Stein R Quaedackers J Bhat NR Dogan HS Nijman RJM Rawashdeh YF. EAU-ESPU pediatric urology guidelines on testicular tumors in prepubertal boys. J Pediatr Urol. (2021) 17:529–33. 10.1016/j.jpurol.2021.06.006 34162520

[4] Geng R Zheng Z Lin Y Li Y Ge G Zhang J. Clinical characteristics and prognostic factors of male yolk sac tumor: a surveillance, epidemiology, and end results program study. World J Urol. (2021) 39:1211–7. 10.1007/s00345-020-03311-y 32562046

[5] Grantham EC Caldwell BT Cost NG. Current urologic care for testicular germ cell tumors in pediatric and adolescent patients. Urol Oncol. (2016) 34:65–75. 10.1016/j.urolonc.2015.06.008 26187598

[6] Song G Xiong GY Fan Y Huang C Kang YM Ji GJ. The role of tumor size, ultrasonographic findings, and serum tumor markers in predicting the likelihood of malignant testicular histology. Asian J Androl. (2019) 21:196–200. 10.4103/aja.aja_119_18 30648671 PMC6413548

[7] O'Neill AF Xia C Krailo MD Shaikh F Pashankar FD Billmire DF. α-Fetoprotein as a predictor of outcome for children with germ cell tumors: a report from the malignant germ cell international consortium. Cancer. (2019) 125:3649–56. 10.1002/cncr.32363 31355926

[8] Balachandran VP Gonen M Smith JJ DeMatteo RP. Nomograms in oncology: more than meets the eye. Lancet Oncol. (2015) 16:e173-80. 10.1016/S1470-2045(14)71116-7 25846097 PMC4465353

[9] Capogrosso P Larcher A Sjoberg DD Vertosick EA Cianflone F Dell'Oglio P. Risk based surveillance after surgical treatment of renal cell. Carcinoma J Urol. (2018) 200:61–7. 29371091 10.1016/j.juro.2018.01.072 PMC6699773

[10] Parker WP Cheville JC Frank I Zaid HB Lohse CM Boorjian SA. Application of the stage, size, grade, and necrosis (SSIGN) score for clear cell renal cell carcinoma in contemporary patients. Eur Urol. (2017) 71:665–73. 10.1016/j.eururo.2016.05.034 27287995 PMC5536178

[11] Ye YL Zheng FF Chen D Zhang J Liu ZW Qin ZK. Relapse in children with clinical stage I testicular yolk sac tumors after initial orchiectomy. Pediatr Surg Int. (2019) 35:383–9. 10.1007/s00383-018-04426-5 30539226

[12] Pan H Shi X Xiao D He J Zhang Y Liang W. Nomogram prediction for the survival of the patients with small cell lung cancer. J Thorac Dis. (2017) 9:507–18. 10.21037/jtd.2017.03.121 28449457 PMC5394048

[13] Liao Y Wang X Zhong P Yin G Fan X Huang C. nomogram for the prediction of overall survival in patients with stage II and III non-small cell lung cancer using a population-based study. Oncol Lett. (2019) 18:5905–16. 10.3892/ol.2019.10977 31788064 PMC6865638

[14] Duan J Xie Y Qu L Wang L Zhou S Wang Y. A nomogram-based immunoprofile predicts overall survival for previously untreated patients with esophageal squamous cell carcinoma after esophagectomy. J Immunother Cancer. (2018) 6:100. 10.1186/s40425-018-0418-7 30285868 PMC6171172

[15] Zhanghuang C Wang J Zhang Z Jin L Tan X Mi T. A web-based prediction model for cancer-specific survival of elderly patients with clear cell renal cell carcinoma: a population-based study. Front Public Health. (2022) 9:833970. 10.3389/fpubh.2021.833970 35310783 PMC8929444

[16] Dias-Santos D Ferrone CR Zheng H Lillemoe KD Fernández-Del Castillo C. The Charlson age comorbidity index predicts early mortality after surgery for pancreatic cancer. Surgery. (2015) 157:881–7. 10.1016/j.surg.2014.12.006 25704415

[17] Amini A Waxweiler TV Maroni PD Kessler ER Cost CR Greffe BS. Survival outcomes of adolescent and adult patients with non-seminomatous testicular germ-cell tumors: a population-based study. J Pediatr Urol. (2016) 12:405. 10.1016/j.jpurol.2016.06.014 27544905

[18] Moch H Cubilla AL Humphrey PA Reuter VE Ulbright TM. The (2016). WHO Classification of tumors of the urinary system and male genital organs-Part A: renal, penile, and testicular tumors. Eur Urol. (2016) 70:93–105. 10.1016/j.eururo.2016.02.029 26935559

[19] Talerman A. The incidence of yolk sac tumor (endodermal sinus tumor) elements in germ cell tumors of the testis in adults. Cancer. (1975) 36:211–5. 1203848 10.1002/1097-0142(197507)36:1<211::aid-cncr2820360122>3.0.co;2-w

[20] Zhou H Zhang Y Song Y Tan W Qiu Z Li S. Marital status is an independent prognostic factor for pancreatic neuroendocrine tumors patients: an analysis of the Surveillance, Epidemiology, and End Results (SEER) database. Clin Res Hepatol Gastroenterol. (2017) 41:476–86. 10.1016/j.clinre.2017.02.008 28416359

[21] Zahoor H Elson P Stephenson A Haber GP Kaouk J Fergany A. Patient characteristics, treatment patterns and prognostic factors in squamous cell bladder cancer. Clin Genitourin Cancer. (2018) 16:e437–42. 10.1016/j.clgc.2017.10.005 29154041

[22] Grady RW. Current management of prepubertal yolk sac tumors of the testis. Urol Clin North Am. (2000) 27:503–8. 10.1016/S0094-0143(05)70097-5 10985149

[23] Frazier AL Hale JP Rodriguez-Galindo C Dang H Olson T Murray MJ. Revised risk classification for pediatric extracranial germ cell tumors based on 25 years of clinical trial data from the United Kingdom and United States. J Clin Oncol. (2015) 33:195–201. 10.1200/JCO.2014.58.3369 25452439 PMC4279239

[24] Pierorazio PM Cheaib JG Patel HD Gupta M Sharma R Zhang A. Comparative effectiveness of surveillance, primary chemotherapy, radiotherapy and retroperitoneal lymph node dissection for the management of early stage testicular germ cell tumors: a systematic review. J Urol. (2021) 205:370–82. 10.1097/JU.0000000000001364 32915080

[25] O'Shea K Tong A Farrelly P Craigie R Cheesman E Shukla R. Management and outcome of pediatric testicular tumors—a 20 year experience. J Pediatr Surg. (2021) 56:2032–6. 10.1016/j.jpedsurg.2021.02.063 33789803

[26] Cornejo KM Frazier L Lee RS Kozakewich HP Young RH. Yolk sac tumor of the testis in infants and children: A clinicopathologic analysis of 33 cases. Am J Surg Pathol. (2015) 39:1121-31. 10.1097/PAS.0000000000000432 25828390

[27] Albers P Albrecht W Algaba F Bokemeyer C Cohn-Cedermark G Fizazi K. European association of urology: guidelines on testicular cancer: update. Eur Urol. (2015) 68:1054–68. 10.1016/j.eururo.2015.07.044 26297604

[28] Gillessen S Sauvé N Collette L Daugaard G de Wit R Albany C. International germ cell cancer classification update consortium. predicting outcomes in men with metastatic nonseminomatous germ cell tumors (NSGCT): results From the IGCCCG Update consortium. J Clin Oncol. (2021) 39:1563–74. Erratum in: *J Clin Oncol*. (2022) 40:2283. 10.1200/JCO.20.03296 33822655 PMC8099402

[29] Seidel C Daugaard G Tryakin A Necchi A Cohn-Cedermark G Ståhl O. The prognostic impact of different tumor marker levels in nonseminomatous germ cell tumor patients with intermediate prognosis: a registry of the International Global Germ Cell Tumor Collaborative Group (G3). Urol Oncol. (2019) 37:809. 10.1016/j.urolonc.2019.07.020 31494007

